# Release of Porcine Sperm from Oviduct Cells is Stimulated by Progesterone and Requires CatSper

**DOI:** 10.1038/s41598-019-55834-z

**Published:** 2019-12-20

**Authors:** Sergio A. Machado, Momal Sharif, Huijing Wang, Nicolai Bovin, David J. Miller

**Affiliations:** 1 Department of Animal Sciences and Institute of Genomic Biology, University of Illinois at Urbana-Champaign, 1207 West Gregory Drive, Urbana, IL 61801 USA; 2 Shemyakin Institute of Bioorganic Chemistry, Moscow, Russia; 3 Present Address: Department of Veterinary Medicine, Western Santa Catarina University, Xanxere, Brazil; 4 Present Address: Department of Obstetrics and Gynecology and Jan and Dan Duncan Neurological Research Institute, Texas Children’s Hospital, Houston, TX 77030 USA; 5 Present Address: Shanghai Jiao Tong University School of Medicine, Shanghai, China

**Keywords:** Extracellular matrix, Glycobiology

## Abstract

Sperm storage in the female reproductive tract after mating and before ovulation is a reproductive strategy used by many species. When insemination and ovulation are poorly synchronized, the formation and maintenance of a functional sperm reservoir improves the possibility of fertilization. In mammals, the oviduct regulates sperm functions, such as Ca^2+^ influx and processes associated with sperm maturation, collectively known as capacitation. A fraction of the stored sperm is released by unknown mechanisms and moves to the site of fertilization. There is an empirical association between the hormonal milieu in the oviduct and sperm detachment; therefore, we tested directly the ability of progesterone to induce sperm release from oviduct cell aggregates. Sperm were allowed to bind to oviduct cells or an immobilized oviduct glycan and then challenged with progesterone, which stimulated the release of 48% of sperm from oviduct cells or 68% of sperm from an immobilized oviduct glycan. The effect of progesterone on sperm release was specific; pregnenolone and 17α-OH-progesterone did not affect sperm release. Ca^2+^ influx into sperm is associated with capacitation and development of hyperactivated motility. Progesterone increased sperm intracellular Ca^2+^, which was abrogated by blocking the sperm–specific Ca^2+^ channel CatSper with NNC 055-0396. NNC 055-0396 also blocked the progesterone-induced sperm release from oviduct cells or immobilized glycan. An inhibitor of the non-genomic progesterone receptor that activates CatSper similarly blocked sperm release. This is the first report indicating that release of sperm from the sperm reservoir is induced by progesterone action through CatSper channels.

## Introduction

Internal fertilization is part of a reproductive strategy that requires male and female gametes to meet in the female reproductive tract. In mammals, fertilization occurs in the upper oviduct. Upon semen deposition in the female reproductive tract, a subpopulation of the ejaculated spermatozoa is transported to the lower oviduct, also called isthmus, where sperm are stored for variable periods, hours to months, depending on the species^1,2^. Retention in the lower oviduct is accomplished in swine by epithelial glycoproteins that contain either of two motifs, a Lewis X trisaccharide (Le^X^) or a biantennary 6-sialylated structure that bind sperm^3,4^. While in the oviduct, sperm complete several maturational changes that allow them to develop fertilizing ability and are referred to collectively as capacitation^5^. Binding to epithelial cells of the isthmus results in functional changes in sperm that enable sperm-egg binding and fertilization even if there is some asynchrony between semen deposition and ovulation. Interaction of sperm with oviduct epithelial cells decreases calcium uptake and suppresses the capacitation-associated increase in protein tyrosine phosphorylation^6–9^. Formation of a “functional sperm reservoir” in the mammalian oviductal isthmus is most likely to allow gradual release of a finite number of competent sperm sub-populations to the fertilization site^10^, lengthening the duration that fertile sperm are provided and reducing the chances of polyspermy^11^. In addition to the peri-ovulatory release of sub-populations of competent sperm, there is evidence for a post-ovulatory release of larger numbers of sperm^12^.

How sperm are released from the oviduct reservoir is controversial. It is not clear whether release is activated by changes in the oviduct fluid, oviduct cells, or sperm. One hypothesis is that sperm detachment is due to a change in oviduct fluid components^13^ or volume. It may also be affected by oviduct peristaltic contractions^13^ or altered oviduct epithelial cell transcription^14,15^. Finally, a change in sperm function may contribute to release. Release may be promoted by the development of hyperactivated motility that may overcome the adhesive forces that otherwise bind sperm to the oviduct epithelium^16–18^. Hyperactivated motility can be activated in human sperm by the sex steroid progesterone^19–22^. The source of the elevated progesterone may be the peri-ovulatory follicle via a counter-current mechanism^23^. The concentration of progesterone is 5–10 fold or more greater in the arterioles near the oviduct^23,24^ and hundreds of times more concentrated in the peri-follicular capillary network^25^ than in systemic circulation.

Progesterone has well-studied effects on human sperm. Progesterone stimulates Ca^2+^ entry in human sperm in a fast and non-genomic fashion^26,27^. Over the last decade, it has been demonstrated that progesterone stimulates Ca^2+^ influx in human sperm by binding to a non-genomic receptor, α/β hydrolase domain-containing protein 2 (ABHD2), a serine hydrolase that depletes membrane 2-arachidonylglycerol, releasing inhibition of sperm Ca^2+^ channels known as CatSper channels^19–21,28–30^. CatSper channels are localized in the principal piece of the sperm flagellum and are essential for male fertility^19–21,29,31^. Functional CatSper channels are necessary for development of human and mouse sperm hyperactivated motility and fertility^22,32–35^. Although there is evidence that CatSper channel subunit genes are expressed in the porcine testis and localized in porcine sperm^36,37^, much less is known of CatSper function outside of human and mouse sperm.

This work was designed to determine the role of progesterone in the release of sperm from the reservoir in the oviduct. Because progesterone is involved in CatSper activation in human and macaque sperm^19,20,22,38,39^, we explored the function of CatSper channels in progesterone-activated porcine sperm release.

## Materials and Methods

### Collection and processing of sperm

Semen was provided by Prairie State Semen Supply and Birchwood Genetics. No live animals were used for these experiments. For each replicate, semen samples were collected from 3–5 different mature boars and diluted in extender. Samples were stored at 16° to 18 °C up to 24 hr prior to use. Three ml of pooled extended semen were washed through a Percoll cushion containing 5.4 ml Percoll, 0.6 ml 10X HBS (1.3 M NaCl, 40 mM KCl, 10 mM CaCl_2_, 5 mM MgCl_2_), and 4 ml dmTALP (2.1 mM CaCl_2_, 3.1 mM KCl, 1.5 mM MgCl_2_, 100 mM NaCl, 0.29 mM KH_2_PO_4_, 0.36% lactic acid, 25 mM NaHCO_3_, 0.6% BSA, 1 mM pyruvic acid, 20 mM HEPES pH 7.3, and sterile filtered) at 800 x g for 10 min. Sperm were washed with 5 ml dmTALP and pelleted for 5 min at 600 x g. Samples with greater than 80% motility were used immediately for experiments. Sperm concentration was determined by hemocytometer and adjusted according to the experiment.

### Collection of oviduct epithelial cells

Oviducts were provided by Rantoul Foods. No live animals were used. For each experiment, the isthmus of 15–20 oviducts were collected from pre- and post-pubertal females and transported in PBS in a sterile 50 ml conical tube on ice. After 2–20 hr on ice, the oviducts were processed at the lab. The isthmus was trimmed and the edge of a microscope slide was used to apply pressure to the outside of the oviduct to strip sheets of oviduct epithelial cells from the isthmus. Epithelial sheets in PBS were transferred to a 15 ml conical tube and centrifuged at 100 × *g* for 1 min. After removing the supernatant, the cells were disaggregated by passage through a 1 ml pipette tip 10 times. After bringing the volume to 15 ml with PBS, the suspension was centrifuged again. The partially disaggregated cells in the pellet were passed through a 22-gauge needle ten times. After adjusting the volume to 12 ml with dmTALP, the cells were divided evenly into three 100-mm tissue culture dishes. Cells were allowed to re-aggregate for 90 min at 39 °C. Spherical aggregates that were 100–150 µm in diameter were selected for experiments.

### Assay of sperm binding to and release from oviduct epithelial cells

Spherical oviduct cell aggregates were selected and washed twice in 100 µL drops of fresh dmTALP. A Stripper Pipette (MidAtlantic Diagnostics, Inc., Mount Laurel, NJ) with a 250 µm internal diameter tip was used to collect oviduct epithelial cell aggregates and wash them. Sperm at a final concentration of 5 × 10^5^ cells/mL were added to 50 µL droplets (total volume) containing oviduct cell aggregates. Sperm and oviduct cell aggregates were pre-incubated at 39 °C for 15 min to allow sperm binding. When testing the necessity for CatSper channels in sperm release, 2 µM of NNC 055–0396, a Ca_v_ channel blocker that blocks CatSper^20^, was also added to this step. Then, the sperm-oviduct cell complexes were transferred in 3 µL to a fresh 47 µL-droplet containing either 800 nM of progesterone or 80 nM of progesterone, pregnenolone, and 17α-OH-progesterone for 30 min at 39 °C or vehicle control. Ten aggregates were added to each droplet in triplicate droplets. After co-incubation, free and loosely attached sperm were removed by washing with 30 µl of dmTALP. Aggregates were transferred onto a microscope slide in a volume of 3 µl. Each droplet with 10 aggregate-sperm complexes was considered an experimental unit for statistical analysis. Images were captured using a Zeiss Axioskop and AxioCam HRc digital camera (Carl Zeiss, Thornwood, NY). The number of sperm bound to the periphery of each aggregate was enumerated and the circumference of the aggregate calculated using AxioVision V 4.5 software (Carl Zeiss, Thornwood, NY). The number of sperm bound per mm circumference was calculated for each aggregate. The average number of sperm bound to the aggregates counted in each droplet was used for statistical analysis.

### Sperm binding and release from 3-O-sulfated lewis X trisaccharide (suLe^X^) coupled beads

Glycan-coated streptavidin-Sepharose High-Performance beads (GE Healthcare Bio-Sciences, Pittsburgh, PA, average diameter of 34 µm) were used to test the ability of sperm to release from an oviduct glycan suLe^X^ in the presence of progesterone. To link the glycan to beads, approximately 60 µg of glycan covalently attached to a biotinylated 30 kDa polyacrylamide core^40^ was incubated with 20 µL of streptavidin-Sepharose beads for 90 min at room temperature. Each molecule of polyacrylamide had 20% glycan and 5% biotin, by molarity^41^. Beads with attached suLe^X^ were washed twice in dmTALP and re-suspended in 100 µl of dmTALP. Once the glycan-coupled beads were ready for use, a 50-µL droplet containing 1.5 million sperm/mL was prepared to receive 1 µL of glycan-coated beads in triplicate droplets. Sperm and beads were co-incubated at 45 min at 39 °C in dmTALP after which the steroid hormones were added. Progesterone (P4), pregnenolone (P5), and 17α-OH-progesterone (17-OHP) at 80 and 800 nM were added to sperm bound to beads. After 30 min of incubation with steroids, the number of sperm bound to the glycan-coated beads was counted. In some experiments, the T channel blocker NCC 55–0396 that inhibits CatSper or a blocker (methoxy arachidonyl fluorophosphonate; MAFP) of the non-genomic progesterone receptor (ABHD2) was added prior to addition of steroid hormone. For each treatment, 25 beads were randomly selected and the total number of bound sperm was enumerated in the triplicate droplets. Sperm that were self-aggregated were not included in the counts. Each experiment was repeated at least 3 times and documented using a Zeiss Axioskop and Axiocam (Zeiss, Thornwood, NY).

### Effects of NNC 55–0396 and methoxy arachidonyl fluorophosphonate on sperm motility

The motility of sperm incubated with 80 nM progesterone, progesterone and CatSper inhibitor NNC 55–0396 (2 µM), 2 µM NNC 55–0396 alone, progesterone and progesterone receptor blocker methoxy arachidonyl fluorophosphonate (2 µM), and methoxy arachidonyl fluorophosphonate (2 µM) alone was assessed using a Hamilton Thorne Semen Analysis CASA system (Hamilton Thorne, Beverly, MA, USA). Sperm were incubated with the treatments at 39 °C in dmTALP for 30 min. For each experimental condition, 5 random fields were evaluated for a minimum total of 100 cells (in each field) in 5 replicates.

### Measurement of intracellular Ca^2+^ in sperm populations

Calcium influx in sperm populations was assessed by a spectrofluorometric assay using a probe that detects intracellular Ca^2+^. These experiments were repeated at least three times. Fluo-4 AM, a Ca^2+^-sensitive reporter, was added at a final concentration of 4 µM to a sperm suspension (5 × 10^6^ sperm/mL in dmTALP) and incubated in the dark for 30 min at room temperature. This incubation was necessary to allow hydrolysis of the acetoxymethyl (AM) ester group by cytoplasmic esterases, enabling Fluo-4 molecules to bind to Ca^2+^. Sperm treated with 80 nM of progesterone or control were incubated at 39 °C and measurements were taken at 0, 15, and 30 min. The actual time of sampling for 0 min was approximately 1–2 min after progesterone addition. In experiments assessing the involvement of CatSper channels during the progesterone-mediated Ca^2+^ influx, either 2 µM of NNC 055–0396 or 500 nM of RU-486 were added 15 min prior to progesterone supplementation. In order to measure strictly intracellular Ca^2+^ signal and account for probe leaking and extrusion from cells, we used 8.4 mM EGTA to chelate extracellular Ca^2+^. Differences in concentrations of free intracellular Ca^2+^ due to binding to Fluo-4 were detected upon argon-ion laser excitation at 494 nm and emission at 516 nm in a QuantaMaster 4CW fluorescence spectrophotometer (Photo Technology International, NJ).

### Statistical analysis

For statistical analysis of sperm binding assay and Ca^2+^ influx is sperm populations, we used SAS software (v. 9.1 SAS Institute, Inc, Cary, NC) to run a one-way analysis of variance using a PROC GLM (General Linear Models) procedure following the general model: *Y*_*ij*_ = *µ* + *α*_*i*_ + *ε*_*ij*_ (where *Y*_*ij*_ is the *j*^th^ sample observation from population *i*; *µ* is the overall mean; *α* is an effect due to population *i*; and *ε* is the random deviation of *Y*_*ij*_ about the *i*^th^ population mean). Results are depicted as means ± SEM. Differences were considered to be significant if p < 0.05 using Tukey’s test for multiple comparisons.

## Results

### Progesterone promotes sperm release from oviduct cell aggregates

**Figure 1:**
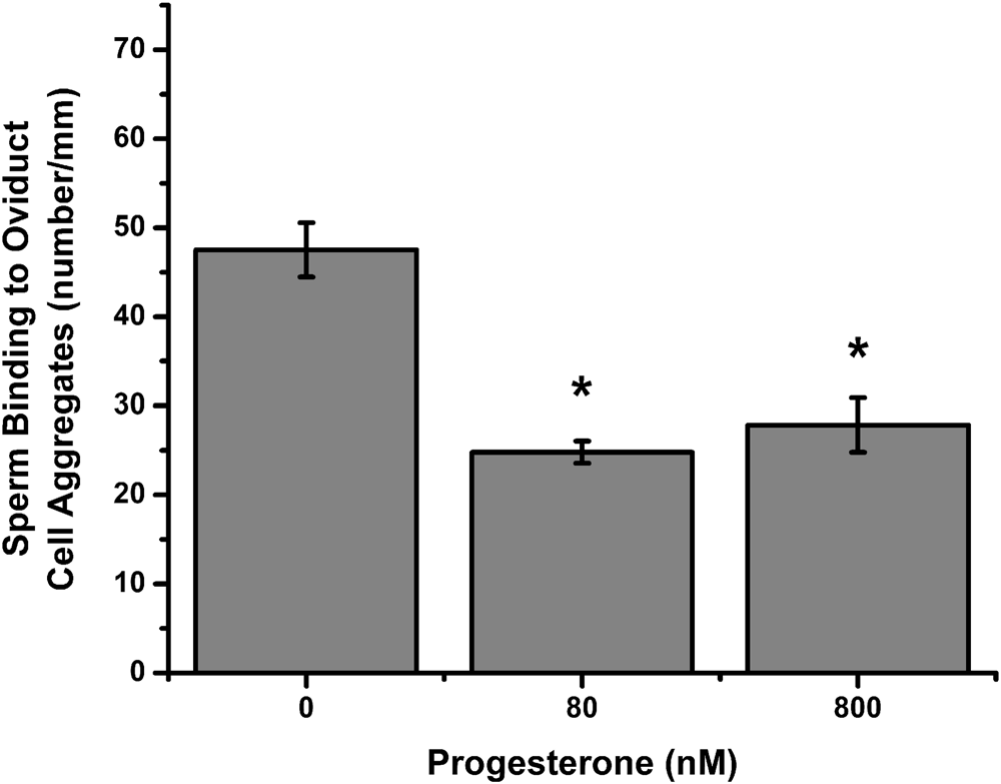
Progesterone promotes sperm release from oviduct cell aggregates *in vitro*. Sperm were allowed to bind to oviduct cell aggregates for 15 min at 39 °C. Sperm-oviduct cell aggregate complexes were treated with 80 and 800 nM of progesterone for 30 min at 39 °C, washed to remove loosely adherent sperm and transferred onto microscope slides for documentation. Progesterone treatment decreased the number of sperm bound to the periphery of epithelial cells when compared to vehicle control. This experiment was repeated four times. Asterisks represent significant differences between treatment and control groups (p < 0.05).

**Figure 2:**
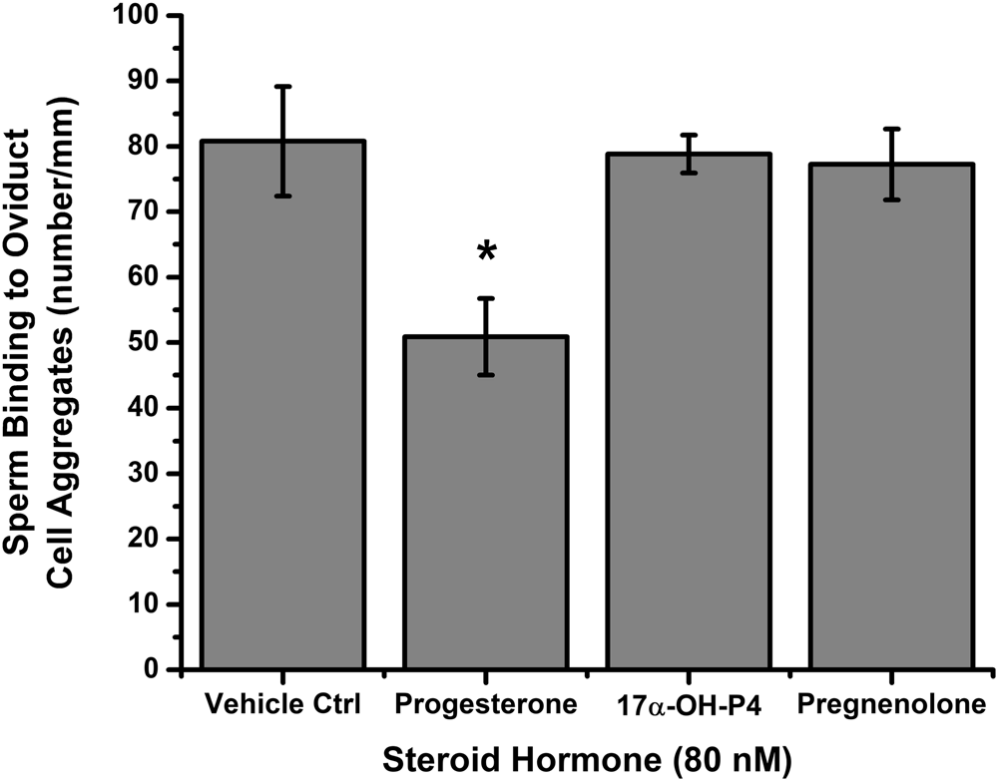
The effect of progesterone on sperm detachment from oviduct cell aggregates is specific. Sperm were allowed to bind to oviduct cell aggregates for 15 min at 39 °C. Sperm-oviduct cell aggregate complexes were treated with 80 nM of either progesterone, 17α-hydroxyprogesterone, or pregnenolone for 30 min at 39 °C. The complexes were washed to remove loosely adherent sperm and transferred onto microscope slides to assess the number of sperm attached to the periphery of the cell aggregates. Progesterone promoted release of sperm from cell aggregates. Pregnenolone and 17α-hydroxyprogesterone, structurally related steroids, did not modify sperm binding. This experiment was repeated three times. The asterisk represents a significant difference among treatments (p < 0.05).

We used an *in vitro* assay to test the ability of progesterone to release porcine sperm from oviduct isthmic cell aggregates. Sperm were allowed to bind the isthmic aggregates for 15 min and then challenged with 80 and 800 nM of progesterone (Fig. 1). Treatment with progesterone stimulated the release of up to 48% of sperm from aggregates within 30 min of addition, compared to the vehicle control group. A higher concentration of progesterone (800 nM) did not stimulate further release of sperm from aggregates. To ensure that progesterone’s effect on sperm release was specific, we treated sperm bound to oviduct aggregate cells with 80 nM of the related steroid hormones pregnenolone and 17α-hydroxyprogesterone. Neither of the two steroid hormones stimulated sperm release; the number of sperm bound to oviduct cell aggregates was not different than the vehicle control (Fig. 2), indicating a specific effect of progesterone on sperm release from oviduct aggregate cells.

**Figure 3:**
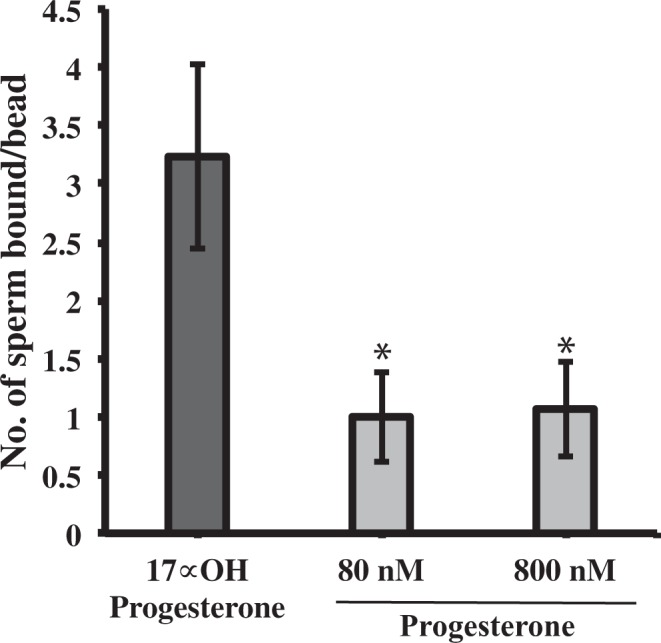
The effect of progesterone on sperm detachment from immobilized 3-O-sulfated Lewis X trisaccharide (suLe^X^). Sperm were allowed to bind to suLe^X^ on beads for 45 min at 39 °C. Sperm bound to suLe^X^-beads were treated with 80 or 800 nM of progesterone or 80 nM, 17α-hydroxyprogesterone for 30 min at 39 °C. The number of sperm bound to beads was enumerated for at least 25 beads per droplet. Progesterone promoted a release of sperm from immobilized suLe^X^, but 17α-hydroxyprogesterone did not. This experiment was repeated three times. The asterisk represents a significant difference compared to 17α-hydroxyprogesterone (p < 0.05).

When added to sperm bound to oviduct cells, progesterone could act on either sperm or oviduct cells. To confirm progesterone was acting on sperm, the oviduct glycan 3-O-sulfated Lewis X trisaccharide (suLe^X^) was covalently attached to biotinylated polyacrylamide and then coupled to streptavidin-beads. Sperm were allowed to bind the beads. Either progesterone or 17α-hydroxyprogesterone was added. Both 80 nM and 800 nM progesterone induced release of 68% of sperm from beads whereas 800 nM 17α-hydroxyprogesterone had no effect (Fig. 3).

**Figure 4:**
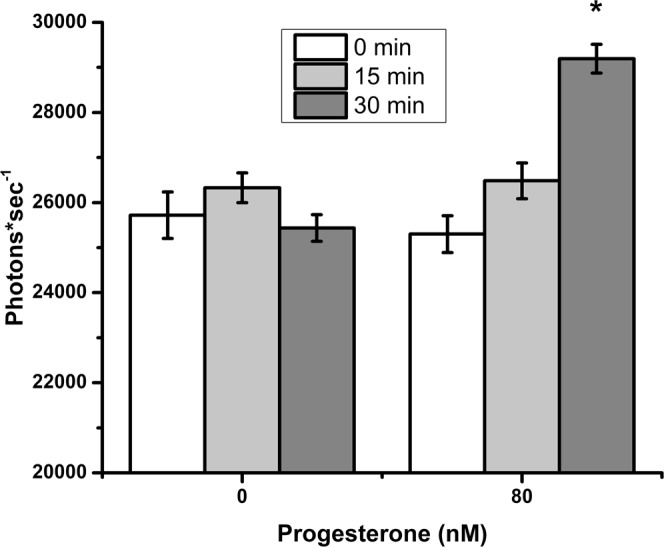
Progesterone stimulates Ca^2+^ influx in sperm. Fluo-4 loaded sperm were incubated in capacitating conditions at 39 °C with 80 nM of progesterone or vehicle. Spectrofluorimetric assessments corresponding to free intracellular Ca^2^ were recorded at 0, 15, and 30 min. Progesterone stimulated significantly more Ca^2+^ influx than the control at 30 min. This experiment was repeated three times. The asterisk represents a significant difference between treatments at the same time-point (p < 0.05).

Because progesterone stimulates Ca^2+^ influx into human sperm^42^ and an increase in intracellular Ca^2+^ is linked to hyperactivation^43^, we assessed intracellular Ca^2+^ in response to progesterone, using the fluorescent Ca^2+^ indicator Fluo-4 AM. The initial measurement (0 min) was taken as soon as possible after adding progesterone (about 1–2 min after progesterone). Although 0 and 15 min incubation with progesterone did not result in significant fluctuations in intracellular free Ca^2+^, treatment of sperm with 80 nM of progesterone for 30 min resulted in a 13% increase in Fluo-4 fluorescence when compared to vehicle controls (Fig. 4).

### CatSper channels are involved in sperm detachment from oviduct cell aggregates

**Figure 5:**
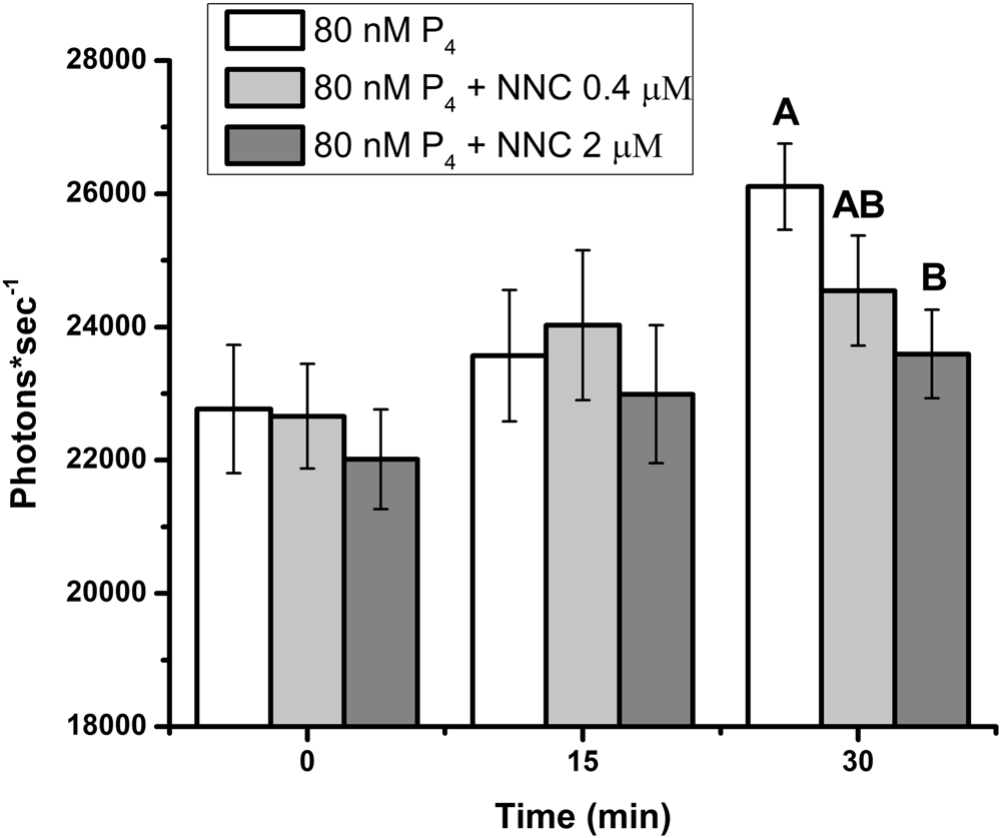
Progesterone (P4)-stimulated Ca^2+^ entry in porcine sperm is dependent on CatSper channels. Sperm loaded with Fluo-4 were incubated in capacitating conditions at 39 °C. All groups were stimulated with 80 nM of P4. Sperm were treated with 0, 0.4, and 2 µM of NNC 55-0396, an inhibitor of CatSper channels. Fluorescence was measured at 0, 15, and 30 min. Inhibition of CatSper channels suppressed the normal rise in Ca^2+^ entry at 30 min stimulated by progesterone. This experiment was repeated three times. Different letters represent significant differences within a time point (p < 0.05).

**Figure 6:**
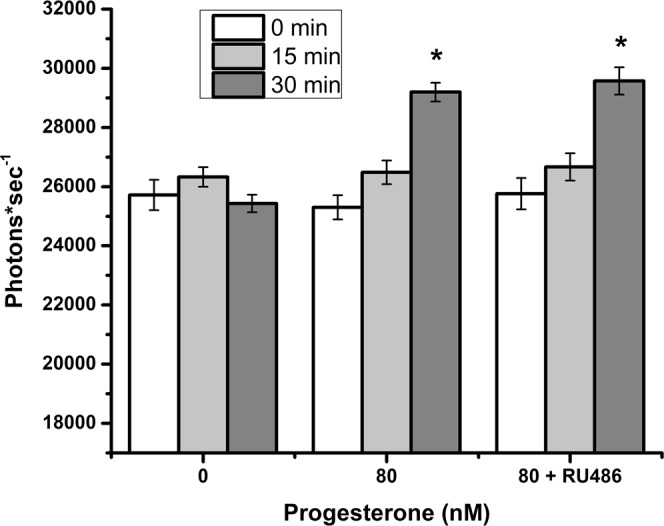
Progesterone-stimulated Ca^2+^ entry in porcine sperm is not dependent on classical progesterone receptors. Sperm loaded with Fluo-4 were incubated in capacitating conditions at 39 °C for up to 30 min. The groups were treated with vehicle control, 80 nM of progesterone, and 80 nM of progesterone plus 500 nM of RU-486 (mifepristone), a genomic progesterone receptor antagonist. Blocking the genomic progesterone receptor did not influence Ca^2+^ entry. The asterisks represent significant differences (p < 0.05) among the groups treated with progesterone and vehicle control. This experiment was repeated three times.

We investigated the possibility that progesterone influences sperm release from oviductal cells by activating CatSper channels. Although CatSper channel activation in porcine sperm is unclear, CatSper channels in human sperm but not mouse sperm are responsive to progesterone^19,20,28,44^ and are essential for male fertility^33,35,39,45–48^. To test the functional importance of CatSper, we used a T-type channel blocker (NNC 055–0396) that abolishes CatSper currents in human sperm^19,20^. We treated the sperm with 0.4 and 2 µM of NNC 055–0396 and free intracellular Ca^2+^ was measured at 0, 15, and 30 min after progesterone addition. Blocking CatSper with 2 µM NNC suppressed the progesterone-induced increase in Fluo-4 fluorescence by 10% when compared to sperm treated with 80 nM progesterone alone (Fig. 5). To rule out the involvement of the genomic progesterone receptor, we used RU-486 (mifepristone), a genomic progesterone receptor antagonist. Mifepristone at a final concentration of 0.5 μM did not affect Ca^2+^ influx (Fig. 6).

**Figure 7:**
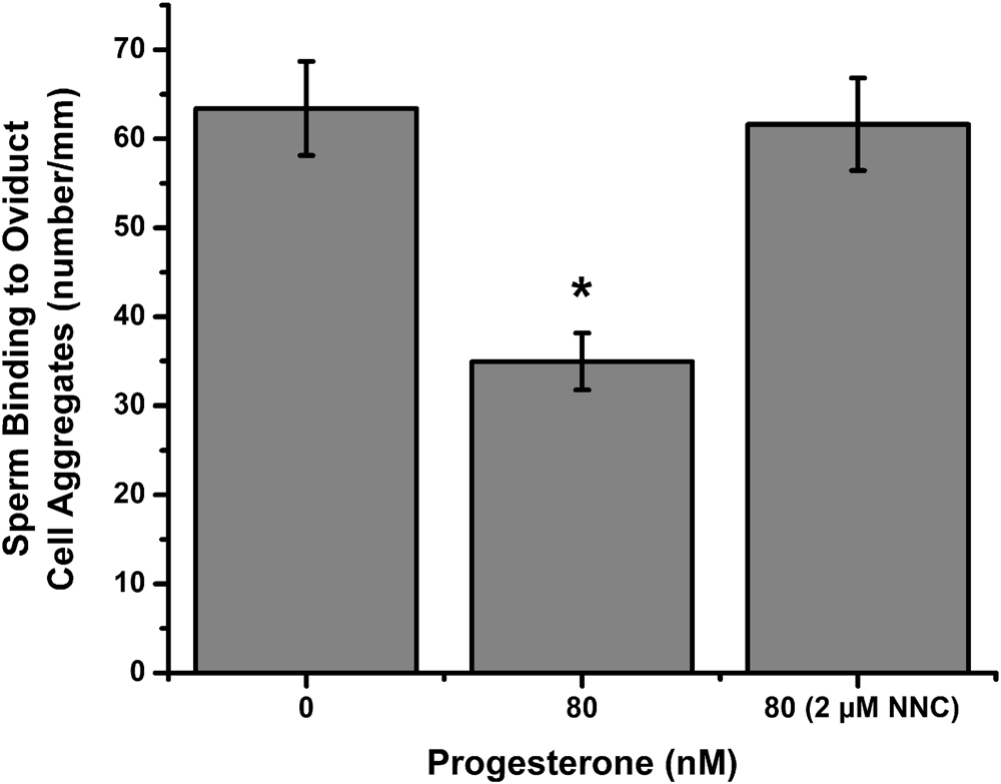
Sperm release from oviduct cell aggregates induced by progesterone is dependent on CatSper channels. Sperm were allowed to bind to oviduct cell aggregates for 45 min at 39 °C and treated with 0 (vehicle control), 80 nM of progesterone, and 80 nM of progesterone plus 2 µM NNC 55-0396 for 30 min at 39 °C. The sperm-aggregate complexes were washed to remove loosely adherent sperm and transferred onto microscope slides to enumerate the number of sperm bound to the periphery of the aggregates. Progesterone reduced the number of sperm bound compared to vehicle control. Blocking CatSper channels abolished the effect of progesterone on sperm release. This experiment was repeated four times. An asterisk represents significant differences among different treatments (p < 0.05).

**Figure 8:**
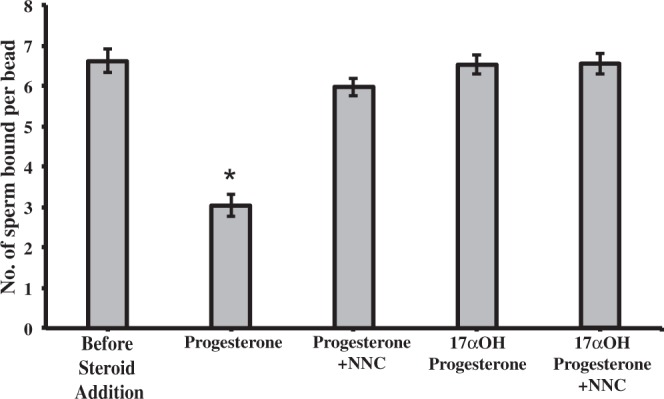
Sperm release from immobilized suLe^X^ is dependent on CatSper channels. Sperm were allowed to bind to immobilized suLe^X^ for 15 min at 39 °C and then treated with 0 (vehicle control), 80 nM of progesterone or 17α-hydroxyprogesterone, with or without 2 µM NNC 55-0396 for 30 min. Sperm bound to beads were enumerated. Progesterone reduced the number of sperm bound compared to vehicle control. Blocking CatSper channels abolished the effect of progesterone on sperm release. This experiment was repeated four times. The asterisk represents a significant difference compared to other treatments (p < 0.05).

**Figure 9:**
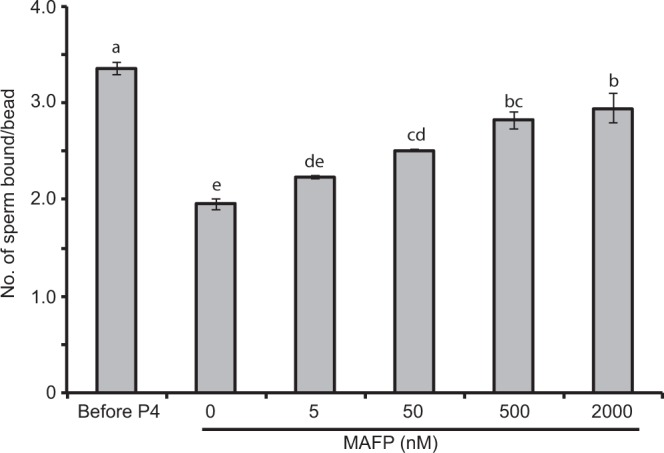
Sperm release from immobilized suLe^X^ is dependent on ABHD2. Sperm were allowed to bind to immobilized suLe^X^ for 15 min at 39 °C and then treated with 80 nM of progesterone, with or without various concentrations of MAFP for 30 min. Sperm bound to beads were enumerated. Progesterone induced release of sperm but release was suppressed by increasing concentrations of MAFP. This experiment was repeated four times. Means with different letters are significantly different (p < 0.05).

Once the association between progesterone and Ca^2+^ influx was established (Figs. 4 and 5), we tested whether functional CatSper channels were involved in sperm release from oviduct cell aggregates. Blocking CatSper with 2 µM NNC 055–0396 inhibited 94% of sperm release induced by progesterone (Fig. 7). The same concentration (2 μM) of NNC 055-0396 also blocked sperm release from suLe^X^-coated beads, demonstrating that the NNC compound was not acting on oviduct cells but rather, sperm (Fig. 8). The non-genomic progesterone receptor in human sperm is α/β hydrolase domain-containing protein 2 (ABHD2), a serine hydrolase that cleaves 2-arachidonylglycerol into glycerol and arachidonic acid^19–21,28–30^. An inhibitor of serine hydrolases, methoxy arachidonyl fluorophosphonate (MAFP), was tested for its ability to abrogate the progesterone-induced sperm release. Concentrations from 5 to 2000 nM of MAFP blocked from 20 to 70% of sperm release (Fig. 9).

One possible explanation for the reduction in sperm release due to NNC or MAFP is that NNC or MAFP reduced the percentage of motile sperm thereby reducing the tension applied to the adhesion between sperm and oviduct glycans and blocking sperm release. To address this possibility, the motility of free sperm exposed to progesterone, NNC and MAFP was assessed. There were no differences in sperm motility characteristics during the 30 min required for sperm release (Table 1). Therefore, sperm release was not blocked due to an effect of NNC and MAFP on sperm motility. This demonstrates that porcine sperm release from isthmic epithelial cells was promoted by progesterone using a mechanism that requires functional CatSper channels.

**Table 1:** Sperm motility parameters 30 min after incubation with progesterone, NNC 55-0396 and methoxy arachidonyl fluorophosphonate (MAFP).

Treatments	P4	P4-NNC	NNC	P4-MAFP	MAFP
Progressive %	35 ± 2.2	31.1 ± 0.7	28.9 ± 0.7	36.4 ± 4.0	32.1 ± 6.0
Motility %	61.6 ± 3.6	58.3 ± 8.5	59.7 ± 8.1	68.8 ± 2.1	62.2 ± 1.1
Rapid %	36.2 ± 8.1	38.8 ± 4.3	37.7 ± 10.1	50.6 ± 5.4	42.3 ± 7.0
VAP μm/sec*	70.2 ± 8.9	75.0 ± 14.1	69.5 ± 13.8	81.9 ± 7.1	75.5 ± 13.4
VSL μm/sec*	46.6 ± 7.0	51.9 ± 14.3	43.2 ± 7.7	52.6 ± 6.9	49.2 ± 11.2
VCL μm/sec*	151.4 ± 9.8	154.6 ± 19.2	149.3 ± 20.8	163.5 ± 17.7	155.8 ± 21.1
ALH μm*	8.6 ± 0.9	8.1 ± 0.5	8.4 ± 1.0	8.9 ± 0.7	8.5 ± 0.6
BCF Hz*	39.2 ± 1.7	37.9 ± 1.8	36.8 ± 2.1	37.4 ± 2.8	37.4 ± 1.7
Straightness %	60.4 ± 5.7	64.2 ± 8.0	58.9 ± 2.0	61.2 ± 3.8	61.4 ± 2.2
Linearity %	29.4 ± 5.0	33.1 ± 7.0	28.8 ± 2.1	31.0 ± 1.5	31.0 ± 2.3
Elongation %	38.4 ± 2.0	42.4 ± 5.0	39.3 ± 2.1	38.8 ± 3.0	40.2 ± 1.9
Area μm/sq	18.4 ± 2.6	17.0 ± 3.1	17.4 ± 5.1	16.0 ± 3.2	16.8 ± 3.0

Motility parameters of sperm incubated with Progesterone (P4), P4 and CatSper inhibitor NNC 55–0396 (P4-NNC), NNC, P4 and P4 receptor blocker MAFP (P4-MAFP), and MAFP alone. Results are means and standard deviations from 5 experiments.

*Path Velocity (VAP) μm/sec, Progressive Velocity (VSL) μm/sec, Track Speed (VCL) μm/sec, Lateral Amplitude (ALH) μm, Beat Frequency (BCF) Hz.

## Discussion

This report documents the importance of progesterone and CatSper channels in the release of porcine sperm from oviduct cells. We demonstrated that progesterone stimulates the detachment of porcine sperm from oviduct cell aggregates *in vitro* through an influx of Ca^2+^ through CatSper channels. These findings indicate that when intracellular free Ca^2+^, a central regulator of sperm function, is increased by addition of progesterone, it promotes sperm release from the oviduct within 30 min.

Although not conclusive, earlier experiments to study sperm release from the isthmus in mammals suggested that ovarian steroids might be involved^12^. There is evidence supporting a role for progesterone, at least some extent, in sperm detachment from the isthmus *in vivo* although the target of progesterone was unclear, either sperm or oviduct cells^11,49–52^. Outside of mammals, there is also evidence that progesterone promotes the release of avian sperm from the storage tubules in the uterovaginal junction^53^. Results herein are the first support for the hypothesis that progesterone acts directly on sperm to release them from oviduct epithelial cells. The progesterone biosynthetic precursor pregnenolone and downstream derivative 17α-hydroxyprogesterone, used as specificity controls, did not cause sperm release. Previous studies demonstrated that estradiol had no effect on sperm binding to oviductal vesicles *in vitro*^50^. Our results are consistent with the findings of an *in vivo* study that reported that injecting progesterone in the isthmic subserosa of gilts in the pre-ovulatory period caused high rates of polyspermic fertilization^51^, presumably by causing a massive release of sperm bound to the oviduct. Although progesterone concentrations are very high during the pre-ovulatory period, especially near the oviduct^25^, how this steroid reaches sperm in the oviduct is unclear. There is a counter-current mechanism that could supply progesterone from the ovary to the oviduct^23,54^. In this model, ovarian steroid hormones diffuse from the ovarian vein to the utero-tubal arteries, redirecting the flow of progesterone toward the oviduct reservoir. Besides the counter-current theory, cumulus-oocyte complexes and detached cumulus cells are likely to synthesize progesterone^55,56^, which, if produced in adequate amounts, could modify the oviductal environment and elicit changes in sperm behavior, including release from the isthmic epithelium.

We demonstrated that nanomolar concentrations of progesterone trigger a rise in intracellular Ca^2+^ in porcine sperm. Although there are many reports of the effect of progesterone on human sperm^44,57,58^, reports of the effects of progesterone on porcine sperm are very limited. Progesterone increased intracellular Ca^2+^ in porcine sperm 0.5 to 1.0 min after addition and stimulated a more gradual secondary increase 30 min later^59,60^. Although the time resolution of our spectrophotometric measurements was insufficient to detect the initial increase, we detected an increase in intracellular Ca^2+^ within 30 min, a time coincident with sperm release (Figs. 1, 3 and 4). Increases in intracellular Ca^2+^ concentrations are commonly associated with a variety of changes that occur during capacitation^61^.

The mechanism by which progesterone increases intracellular Ca^2+^ through CatSper is best described in human sperm. Progesterone binds to ABHD2, a serine hydrolase that, after progesterone binding, removes CatSper inhibitors allowing the channel to open^28^. But in mouse sperm, CatSper is activated by an increase in intracellular pH^62,63^. This mere change in pH appears inadequate to activate human CatSper. There is also evidence that other voltage-gated Ca^2+^ channels (Ca_V_2.3) have a role in mouse sperm function^64^. Outside of human and mouse sperm, the function of CatSper is less clear. Bovine and equine sperm hyperactivation is promoted by intracellular alkalinization-induced activation of Ca^2+^ influx, presumably occurring through CatSper^65,66^. We tested the functional importance of CatSper in porcine sperm release by blocking the progesterone-mediated Ca^2+^ entry in sperm using NNC 55-0396, a T-type calcium channel inhibitor^19,20,67^ and by blocking ABHD2. Blocking CatSper was sufficient to suppress the Ca^2+^ influx induced by progesterone (Fig. 5). Progesterone-mediated Ca^2+^ entry in sperm was CatSper-specific; mifepristone, a classical progesterone receptor antagonist, did not modify Ca^2+^ influx (Fig. 6). These results suggest that the Ca^2+^ entry in porcine sperm that is influenced by progesterone is occurring by activation of CatSper channels, which are essential for the development of sperm hyperactivated motility and sperm fertilizing ability^34,68^.

Not only did blocking CatSper abrogate the increase in intracellular Ca^2+^, it completely blocked sperm release from oviduct cells and an immobilized oviduct glycan. Similarly, inhibition of ABHD2 also suppressed sperm release. These results suggested that sperm hyperactivation is critical and sufficient for sperm release, in contrast to a previous report that addition of a glycosaminoglycan in the medium was necessary for release^13^. Our results also suggest that oviduct fluid flow or oviduct peristaltic contractions are not necessary for sperm release because release occurred from isolated epithelial cells and beads in droplets that have minimal fluid flow (Fig. 1). The inhibition of sperm release from oviduct cells was not due to reduction in the percentage of motile sperm or other motility characteristics evaluated by CASA (Table 1). We also did not detect changes in hyperactivation, as determined by CASA, in response to progesterone. But it is challenging for CASA to discern traits in porcine sperm that are associated with hyperactivation in other species (i.e. changes in BCF, ALH, straightness and linearity) because of the very asymmetrical full-type hyperactivated motility that porcine sperm display^69,70^.

It is highly unlikely that NNC 55-0396, a T-type calcium channel inhibitor, prevented sperm release by acting on oviduct cells rather than sperm. Although ciliated oviduct cells have TRPV4 channels that are likely affected by the NNC compound, TRPV4 channels of oviduct ciliated cells are activated by high fluid viscosity^71^. But NNC 55-0396 blocked sperm release under conditions in which fluid viscosity was not changed. Furthermore, the NNC compound blocked sperm release from an immobilized oviduct cell glycan, suLe^X^ in the absence of oviduct cells. Finally, an inhibitor of ABHD2 also blocked release from the immobilized oviduct cell glycan.

Our data support the model that release of porcine sperm from oviduct isthmic cells is activated by progesterone and requires ABHD2 and CatSper channels. Increasing progesterone concentrations in the sperm reservoir might be one of the signals that accompanies ovulation and facilitates release of sperm from the oviduct epithelium so that they can be freed to fertilize oocytes. These are the first results showing that progesterone is sufficient to release mammalian sperm from oviduct epithelial cells. This work helps explain the intricate communication necessary for successful mammalian fertilization.
